# Revision of the Afrotropical genus *Fainia* Zumpt, 1958, with notes on the morphology of Rhiniidae subfamilies (Diptera, Oestroidea)

**DOI:** 10.3897/zookeys.1033.58539

**Published:** 2021-04-22

**Authors:** Arianna Thomas-Cabianca, Anabel Martínez-Sánchez, Martin H. Villet, Santos Rojo

**Affiliations:** 1 Departamento de Ciencias Ambientales y Recursos Naturales, Universidad de Alicante, Carretera San Vicente del Raspeig s/n, Aptdo 99 E-03080, San Vicente del Raspeig, Alicante, Spain Universidad de Alicante Alicante Spain; 2 Department of Zoology & Entomology, Rhodes University, PO Box 94, Makhanda 6140, South Africa Rhodes University Grahamstown South Africa

**Keywords:** Apomorphies, identification key, nose flies, taxonomy

## Abstract

The taxonomy and diversity of *Fainia* Zumpt, 1958, an exclusive Afrotropical genus, had not been reviewed recently. The genus included six nominal species, but the status of several of them was debated. Identification of most *Fainia* species depends on characters of the male terminalia; females are poorly known and, in several cases, are not adequately diagnosed. We conducted a taxonomic revision of the genus and generated identification tools. Based on the study of type material and specimens available in entomological collections in Africa and Europe, we recognise here three of the six species as valid (*F. albitarsis* (Macquart, 1846), *F. elongata* (Bezzi, 1908) and *F. inexpectata* Zumpt, 1973). We also provide an identification key to both sexes, redescriptions of the species, updated distribution records and high resolution photographs of males’ and females’ habitus and male terminalia. The description of *Fainia kagerana* Lehrer, 2007a **nom. nud.** is an invalid nomenclatural act in terms of ICZN Article 13.1.1. Based on examinations of their holotypes, *F. sambura* Lehrer, 2008 **syn. nov.** is proposed as a junior synonym of *F. albitarsis*; *F. kirinyaga* Lehrer, 2007b **syn. nov.** is proposed as a junior synonym of *F. inexpectata*; and *Fainia giriama* Lehrer, 2007b is moved from the genus *Fainia* to the genus *Rhinia* Robineau-Desvoidy, as *Rhinia giriama* (Lehrer, 2007b) **comb. nov.**. We propose two apomorphies that support the status of the subfamily Rhiniinae.

## Introduction

The fly family Rhiniidae, distributed in the Afrotropical, Australasian, Oriental and Palaearctic Regions, includes about 376 described species in 30 genera that have traditionally been placed in two subfamilies: Cosmininae and Rhiniinae (Malloch 1926; Peris 1952, 1992; Pont 1980; Pape et al. 2011). Recent molecular evidence shows that Cosmininae is paraphyletic, separating *Sumatria* Malloch from the rest of the traditional concept of the subfamily Cosmininae, while Rhiniinae is monophyletic (Buenaventura et al. 2020). Rhiniinae includes four genera from the Afrotropical Region: *Fainia* Zumpt, 1958, *Rhinia* Robineau-Desvoidy, *Stomorhina* Rondani and *Vanemdenia* Peris (Zumpt 1962; Kurahashi and Kirk-Spriggs 2006) and is generally recognised by the presence of a long, pectinate antennal arista; reduced dorsal thoracic chaetotaxy; acrostichal and dorsocentral setae that are restricted to the prescutellars pairs; a bare suprasquamal ridge; and a bare proepisternum (Peris 1952, 1992; Zumpt 1958, 1962).

The genus *Fainia* was erected by Zumpt (1958) after the study of two Afrotropical species then assigned to *Idiella* Brauer & Bergenstamm. He distinguished them from the Oriental/Palaearctic *Idiella* species by the unusual shape of the fifth sternite lobes and the fused cerci of the male terminalia. Later, Lehrer (2007a) proposed the subfamily Fainiinae, based on the morphologies of the phallus and the fifth and sixth sternites of *Fainia*, which differed considerably from those of other Afrotropical genera of Rhiniinae. Recent phylogenetic studies place *Fainia* within Rhiniinae as sister-taxon to *Rhinia* (Buenaventura et al. 2020). Prior to that study, *Fainia* comprised six nominal species: *F. albitarsis* (Macquart, 1846), *F. elongata* Bezzi, 1908, *F. inexpectata* Zumpt, 1973, *F. kirinyaga* Lehrer, 2007b, *F. giriama* Lehrer, 2007b and *F. sambura* Lehrer, 2008. However, species of Calliphoridae, Polleniidae and Rhiniidae described by Lehrer need to be revised carefully (e.g. Rognes 2005, 2009, 2011, 2012; Gisondi et al. 2020).

There is very little information on the diversity, biology and distribution of the Rhiniidae. The life cycle and, in particular, the habits and larval morphology are unknown for most of the species (Cuthbertson 1933, 1934; Kurahashi and Kirk-Spriggs 2006; Peris 1952; Zumpt 1958). What is known is limited to a few species that are restricted to specific geographic regions. In general, some species have a strong association with natural environments; adults are flower visitors and are thought to be important pollinators; and some species seem to have a close relationship with termites (Arce et al. 2019; Kurahashi and Kirk-Spriggs 2006; Ferrar 1987).

We present a morphological revision of *Fainia*, including a taxonomic study; an update of nomenclature with morphological considerations of key characters; an identification key; redescriptions; and high quality photographs of males’ and females’ habitus and male terminalia. We propose two apomorphic characters of the phallus that allow diagnostic differentiation of Rhiniinae.

## Material and methods

This study is based in the examination of 59 specimens housed in 10 entomological collections. Available type specimens of the species were examined. The following acronyms were used in the text for the institutions housing the specimens that were examined:

**BMSA**Department of Entomology, National Museum, Bloemfontein, South Africa;

**CEUA** Entomological Collection, University of Alicante, Alicante, Spain;

**DMSA**Durban Natural Science Museum, Durban, South Africa;

**MNHN**Muséum National d’histoire Naturelle, Paris, France;

**MZSUR** Zoology Museum, La Sapienza University of Rome, Rome, Italy;

**NMSA**KwaZulu-Natal Museum, Pietermaritzburg, South Africa;

**SAMC**Iziko South African Museum, Cape Town, South Africa;

**SMNHTAU (TAUI)**Steinhardt Museum of Natural History, Tel Aviv University, Tel Aviv, Israel;

**ZMHB**Museum für Naturkunde, Leibniz-Institut für Evolutions- und Biodiversitätsforschung, Berlin, Germany;

**ZMUC**Zoologisk Museum, University of Copenhagen, Copenhagen, Denmark.

### Morphology and terminology

**Figure 1.**
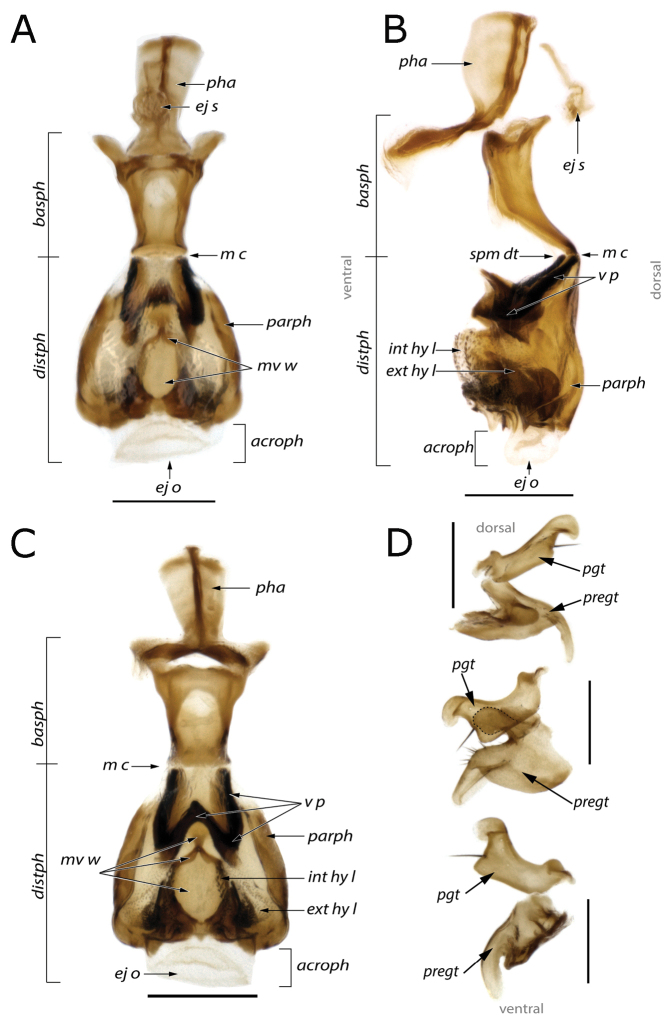
Phallus of *Fainia albitarsis* (Macquart, 1846) **A** dorsal view **B** lateral view **C** ventral view **D** postgonite and pregonite in lateral view. Abbreviations: *acroph* – acrophallus; *basph* – basiphallus; *distph* – distiphallus; *ej e* – ejaculatory sclerite; *ej o* – ejaculatory opening; *ext hy l* – external (distal) hypophallic lobe; *int hy l* – internal (proximal) hypophallic lobe; *m c* – membranous connection; *m w* – mid-ventral wall; *parph* – paraphallus; *pgt* – postgonite; *pha* – phallapodeme; *pregt* – pregonite; *spm dt* – sperm duct; *v p* – ventral plate. Scale bars: 0.2 mm.

**Figure 2.**
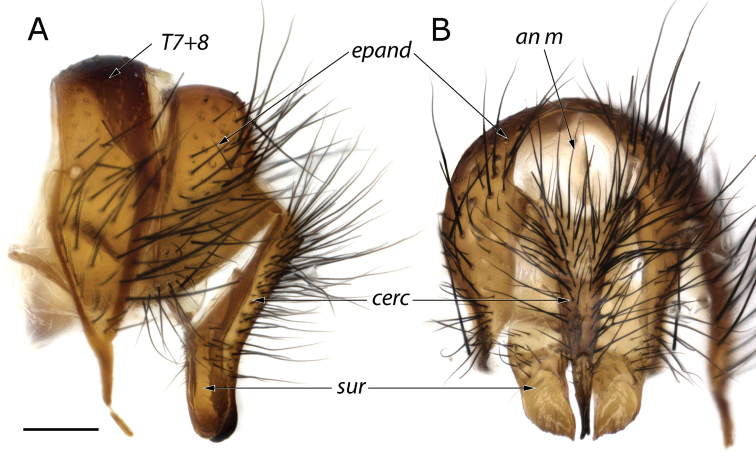
Details of the epandrial complex of *Fainia albitarsis* (Macquart, 1846) **A** lateral view **B** posterior view. Abbreviations: *an m* – anal membrane; *cerc* – cercus; *epand* – epandrium; *sur* – surstylus; *T7+8* – tergite 7 + 8. Scale bar: 0.2 mm.

**Figure 3.**
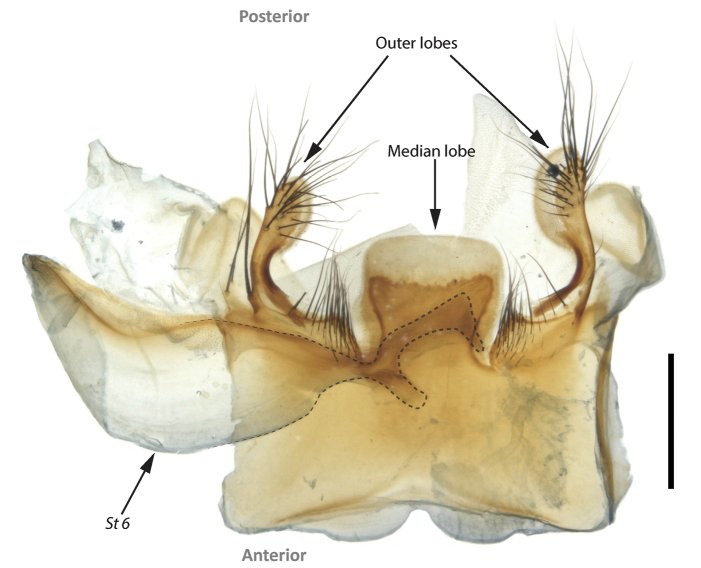
Sternite 5 of *Fainia elongata* (Bezzi, 1908). Abbreviation: *St6* – sternite 6. Scale bar: 0.5 mm.

Morphological characters and terminology follow Cumming and Wood (2017). Male terminalia characters are based on Buenaventura and Pape (2018), Cerretti et al. (2014) and Rognes (1991, 2002, 2009, 2013). Characters of the Rhiniidae male terminalia and fifth sternite are illustrated in Figs 1–3.

### Preparation and taxonomic revision of specimens

**Figure 4.**
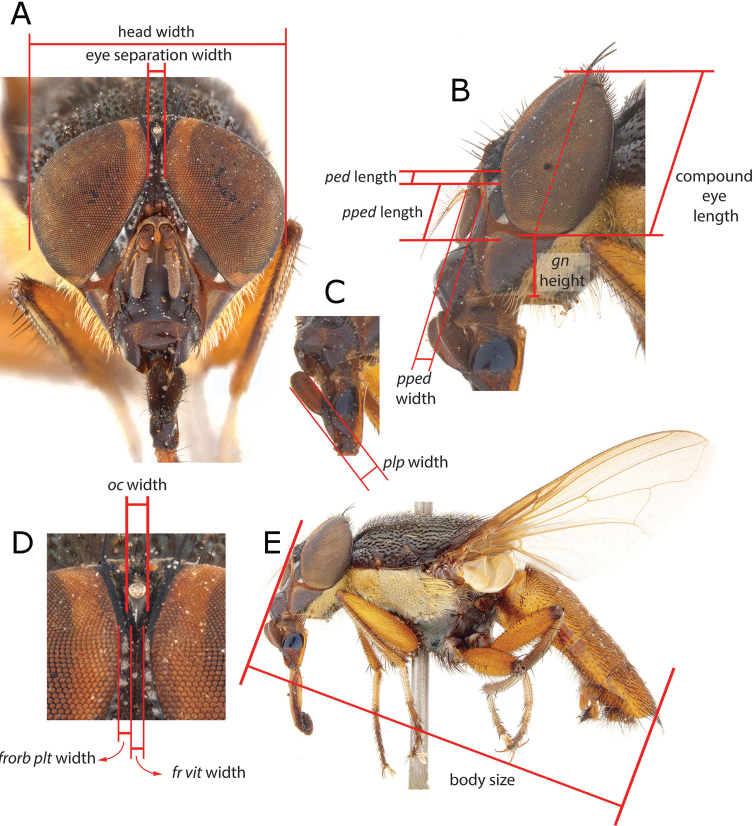
Specification of the characters measured (species = *Fainia albitarsis* (Macquart, 1846)) **A** head in frontal view showing maximum head width and eye separation width **B** head in lateral view showing maximum eye height (spotted red line), maximum gena height, pedicel length and postpedicel length and width **C** palpus in lateral view showing palpus width **D** upper part of head in frontal view showing frontal vitta width, fronto-orbital plate width and anterior ocellus width **E** body size in lateral view. Abbreviations: *fr vit* – frontal vitta; *frorb plt* – fronto-orbital plate; *gn* – gena; *oc* – anterior ocellus; *ped* – pedicel; *plp* – palpus; *pped* – postpedicel.

Pinned and ethanol-preserved specimens were examined using stereomicroscopes (Leica M80 and Leica MZ95) with an ocular micrometer and external LED illumination. Identifications and reidentifications were made following Peris (1952), Zumpt (1958, 1973) and Lehrer (2007a, 2007b, 2008, 2011). Females were identified by morphological comparison with males and the species’ descriptions and corroborated using DNA barcodes (Thomas-Cabianca et al., unpublished). Male terminalia were dissected following Rognes (2009) and Cerretti and Pape (2012), stored in small plastic microvials filled with glycerine and pinned or preserved together with their respective specimens. Measurements made in this study are summarised in Fig. 4.

### Identification tools

The taxonomic key and descriptions were based on a morphological character matrix built using the DELTA (DEscription Language for TAxonomy) software (Dallwitz 1980a, b, Dallwitz et al. 1999) and building on leads from the DELTA-IntKey module (Dallwitz et al. 2000).

### Composite macro-microphotographs

Adult specimens were photographed using a Canon-EOS 6D reflex camera with Canon MP-E 65 mm f/2.8 1–5 lens (ISO 200, f/5.6-9, V:1/160) installed on a copy table with an automatic macro-metric rail and external artificial light or using a Canon-EOS 7D camera with K2-P1CF2 lenses and a P-51 Camlift controller, version 2.8.0.0 (Copyright Roy Larimer/Dun.inc.2014). Photographs included habitus (dorsal and lateral views), head (frontal and lateral views) and abdomen (dorsal and lateral views). Additional photographs of important morphological structures were also taken. Between 15 and 60 high-resolution pictures (in RAW or TIFF format) were taken to cover all of the focal planes needed for focus stacking. Male terminalia were photographed using a stereomicroscope with an integrated Leica M205C camera and coupled DFC450 camera and a Leica Z16AP0A macroscope with coupled Leica DFC490 camera. Photographs included the epandrium, cerci and surstyli (dorsal and lateral view), phallus (lateral, dorsal and ventral view), postgonite and pregonite (lateral view), ejaculatory sclerite (if available) and fifth sternite. Images were processed using Adobe Photoshop Lightroom CS6, stacked with Zerene Stacker, edited with Adobe Photoshop CS6 and measured with IMAGEJ.

### Information provided

For each species we provide: valid name, synonyms, diagnosis (included in the identification key), type locality and type repository (including primary types), distribution, biology, redescriptions of male and female, material examined and photographs. Previously unpublished records obtained from the material examined are indicated by an asterisk (*). Some countries are marked with a ‘?’ when the report was a museum database record that showed discrepancies after our examination of the relevant specimen(s).

### Citation of specimen label data

Label data of the type material reviewed were recorded verbatim, with information for each line separated by a virgule (/) and labels separated by a double virgule (//). For non-type specimens, the ‘material examined’ section includes selected information from specimen labels, here presented as: country, province, number of individuals per sex, locality, geographical coordinates, reported elevation, date(s) and collector(s) (leg.), collection method, biological or environmental information, determiner (det.) and date of identification; repository and specimen code (provided by the institution); and male terminalia slide code. Abbreviations used: BECE = Boyekoli Ebale Congo Expedition, HT = holotype, PT = paratype, TS = ♂ terminalia slide, TSP = terminalia slide preparation, KR = Knut Rognes identification database number, ♂ = male, ♂♂ = males, ♀ = female, ♀♀ = females.

## Results

### Fainia

Taxon classificationAnimaliaDipteraRhiniidae

Zumpt, 1958

A6498F13-5E29-5630-8457-554DC5A6FCE8

Figs 1 , 2 , 3 , 4 , 5 , 6 , 7 , 8 , 9 , 10 , 11 , 12 , 13

#### Type species.

*Idia albitarsis* Macquart, 1846, by original designation.

#### Diagnosis.

**Figure 5.**
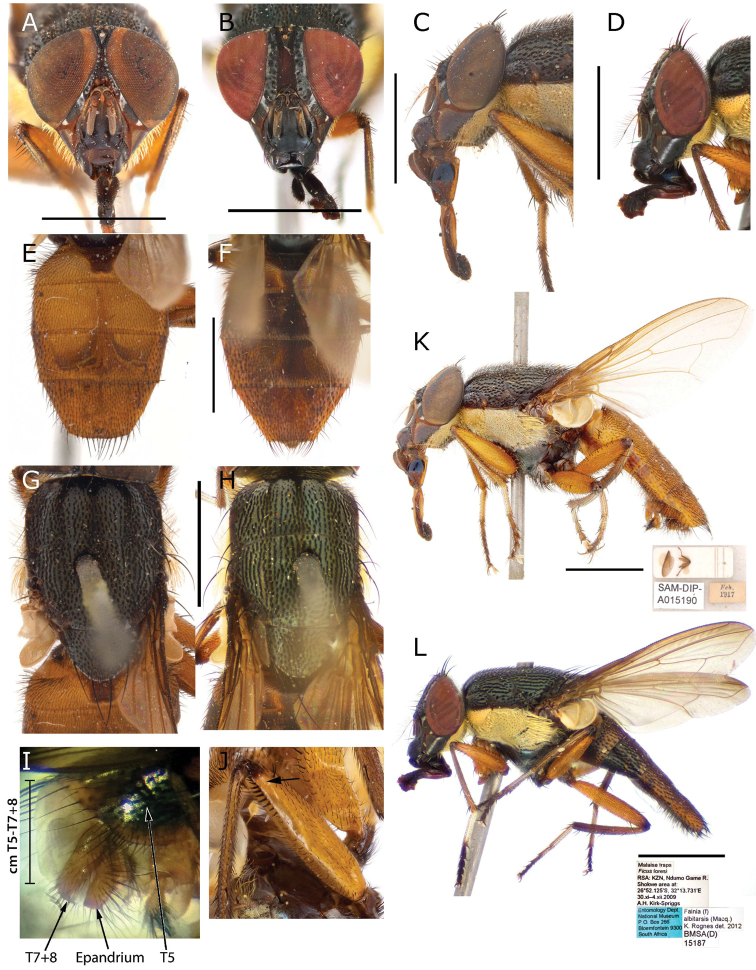
*Fainia albitarsis* (Macquart, 1846), general body views of male (SAM DIP A015190) and female (BMSA (D) 15187) **A, C, E, G, I–K** male **A** head in frontal view **C** head in lateral view **E** abdomen in dorsal view **G** thorax in dorsal view **I** retractile membrane connecting *T5* and *T7+8***J** mid-femur, showing posteroventral row of closely spaced spine-like setae distally **K** lateral habitus and labels **B, D, F, H, L** female **B** head in frontal view **D** head in lateral view **F** abdomen in dorsal view **H** thorax in dorsal view **L** lateral habitus and labels. Abbreviations: *cm T5-T7+8* – connective membrane between tergite 5 and 7+8, *T5* – tergite 5, *T7+8* – tergite 7+8. Scale bars: 2 mm.

**Figure 6.**
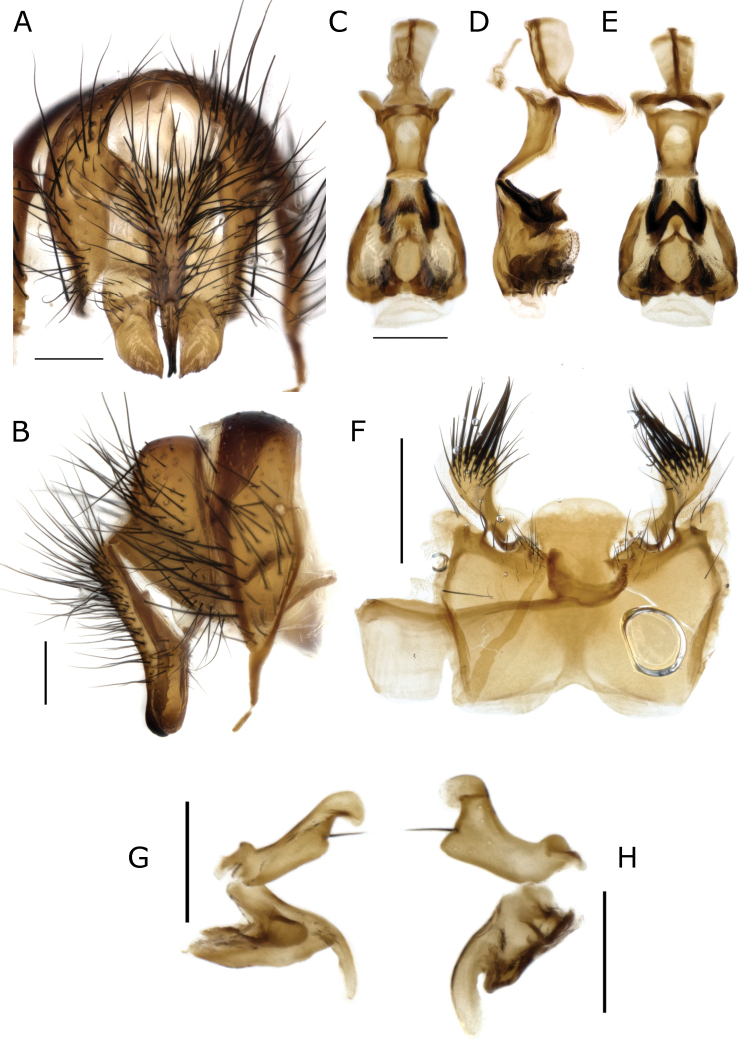
*Fainia albitarsis* (Macquart, 1846), male terminalia (BMSA (D) 30066) **A, B** epandrial complex and tergite 7+8 in dorsal (**A**) and lateral (**B**) view **C–E** phallus in dorsal (**C**), lateral (**D**) and ventral (**E**) view **F** sternite 5 in ventral view and **G, H** postgonite (upper) and pregonite (lower) in lateral-external (**G**) and lateral-internal view (**H**). Scale bars: 0.2 mm.

**Figure 7.**
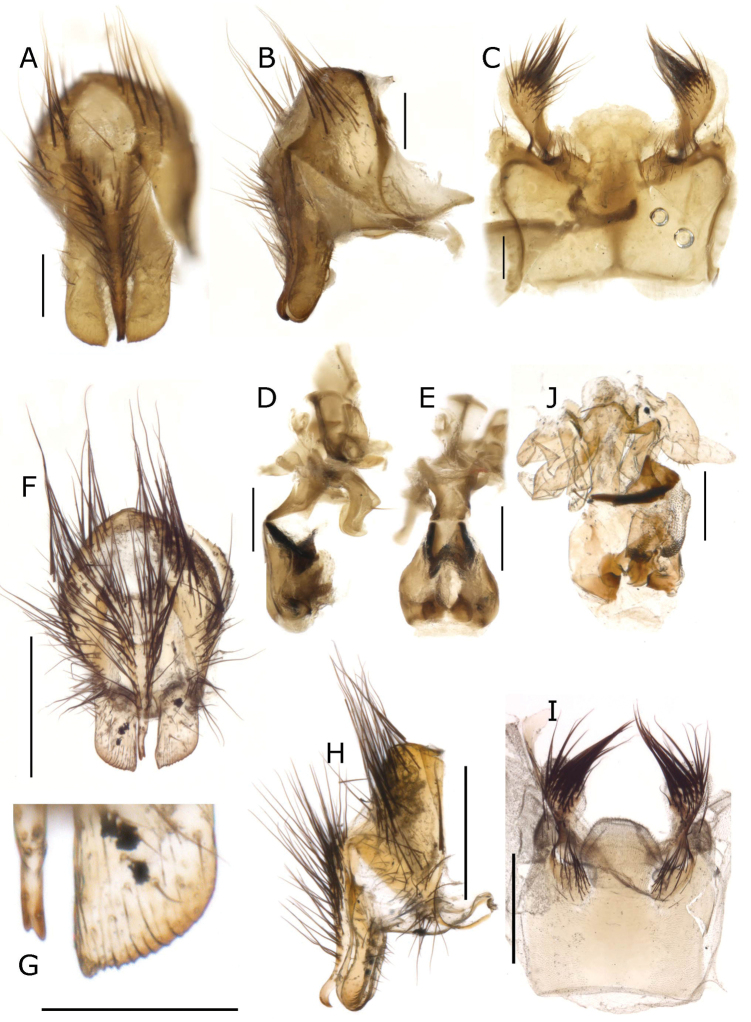
*Fainia kagerana* Lehrer, 2007a nom. nud. (SMNHTAU (TAUI) 318988) and *Fainia sambura* Lehrer, 2008 holotype (SMNHTAU (TAUI) 318990), male terminalia **A–E***F. kagerana***A, B** epandrial complex in dorsal (**A**) and lateral (**B**) view **C** sternite 5 in ventral view and **D, E** phallus in lateral (**D**) and ventral (**E**) view **F–J***F. sambura***F–H** epandrial complex in dorsal view (**F**), details of surstylus, showing serrations along distal margin (**G**) and lateral view (**H**) and details in **I** sternite 5 in ventral view **J** phallus view. Scale bars: 0.2 mm.

***Head*.** Arista dorsally pectinate, male eyes separated at narrowest point by less than width of ocellar triangle. ***Thorax*.** Anepisternum with two upper posterior setae and dense yellow microtomentum; katepisternum with or without yellow microtomentum; thoracic chaetotaxy reduced (presutural acrostichal, dorsocentral and intra-alar absent and postsutural acrostichal and dorsocentral setae restricted to prescutellars). ***Legs*.** First tarsomeres always light cream-coloured; fore tibia without submedial posteroventral setae; hind tibia with 2–3 anterodorsal setae as long as tibial diameter, but not forming a distinct row (Fig. 10I). Male mid femur with a distal posteroventral row (ctenidium) of closely spaced spine-like setae (Fig. 5J; this character is also found in some *Stomorhina* species, such as *S. apta* Curran, 1931 and *S. malobana* (Lehrer, 2007c)). ***Wing*.** Cell *r_4+5_* always open. ***Male terminalia***. Tergites 5 and 7+8 connected by a long retractile membrane (Fig. 5I), tergite 6 not sclerotised, spiracle 6 present, cerci fused (Figs 2B, 6A, 7A, F, 9A, 11A, 12A) and sternite 5 divided into three posterior lobes (Figs 3, 6F, 7C, I, 9F, 11F, 12F).

#### Redescription.

**Figure 8.**
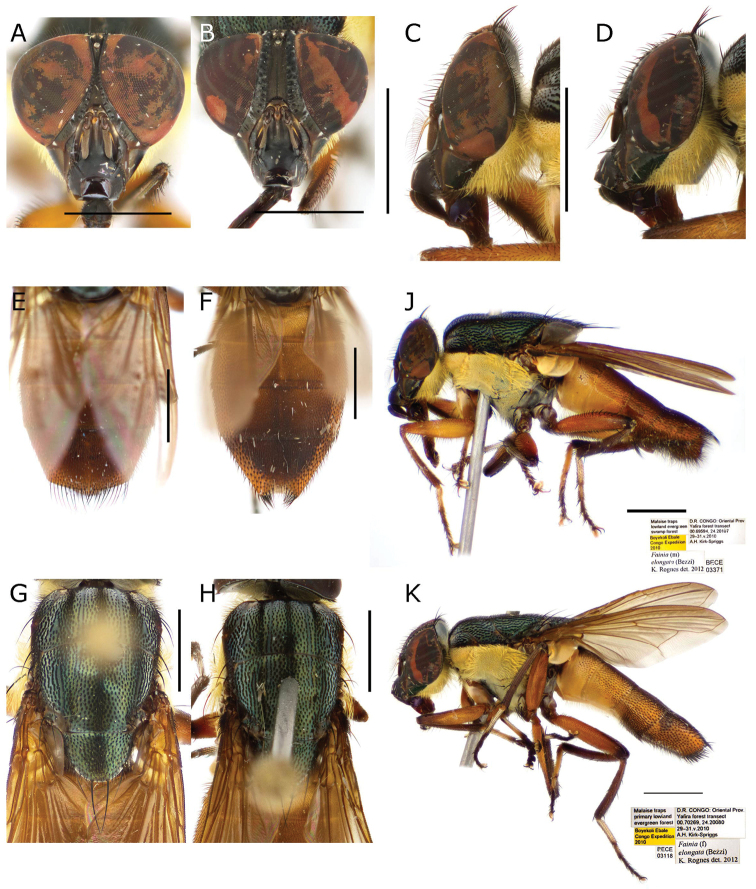
*Fainia elongata* (Bezzi, 1908). General body views of male (BMSA-BECE 03371) and female (BMSA-BECE 03118) **A, C, E, G, J** male **A** head in frontal view **C** head in lateral view **E** abdomen in dorsal view **G** thorax in dorsal view **J** lateral habitus and labels **B, D, F, H, K** female **B** head in frontal view **D** head in lateral view **F** abdomen in dorsal view **H** thorax in dorsal view **K** lateral habitus and labels. Scale bars: 2 mm.

**Figure 9.**
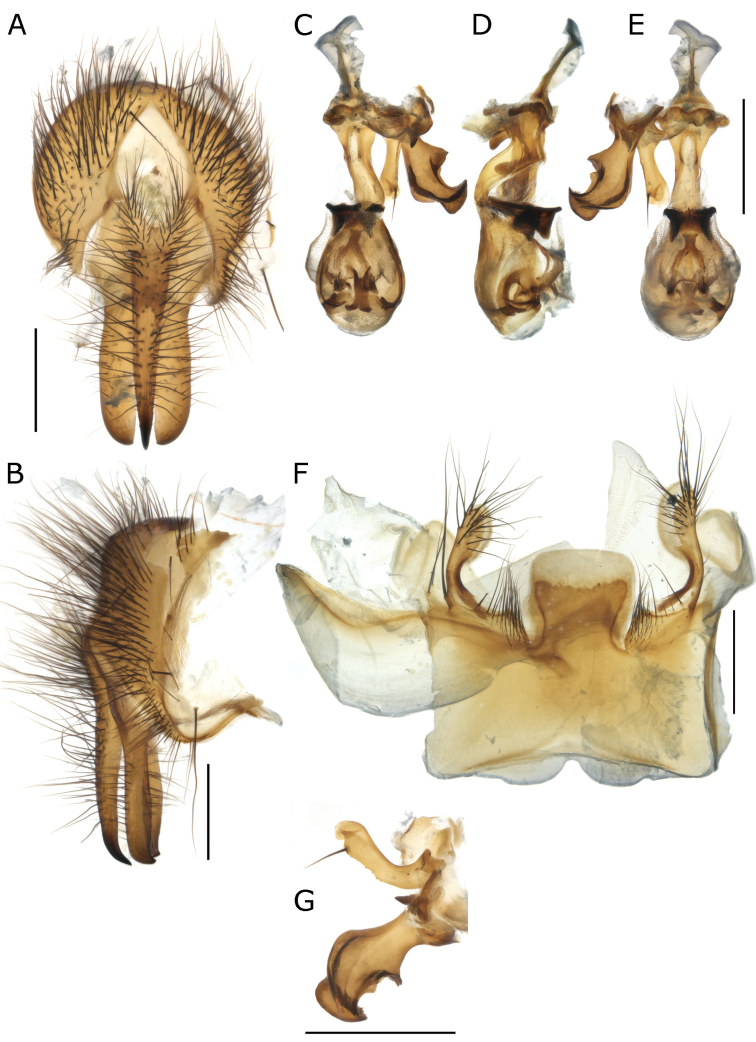
*Fainia elongata* (Bezzi, 1908). Male terminalia (BMSA-BECE 03371) **A, B** epandrial complex in dorsal (**A**) and lateral (**B**) view **C–E** phallus in dorsal (**C**), lateral (**D**) and ventral (**E**) view **F** sternite 5 in ventral view and **G** postgonite (upper) and pregonite in lateral-external view (**G**). Scale bars: 0.2 mm.

**Figure 10.**
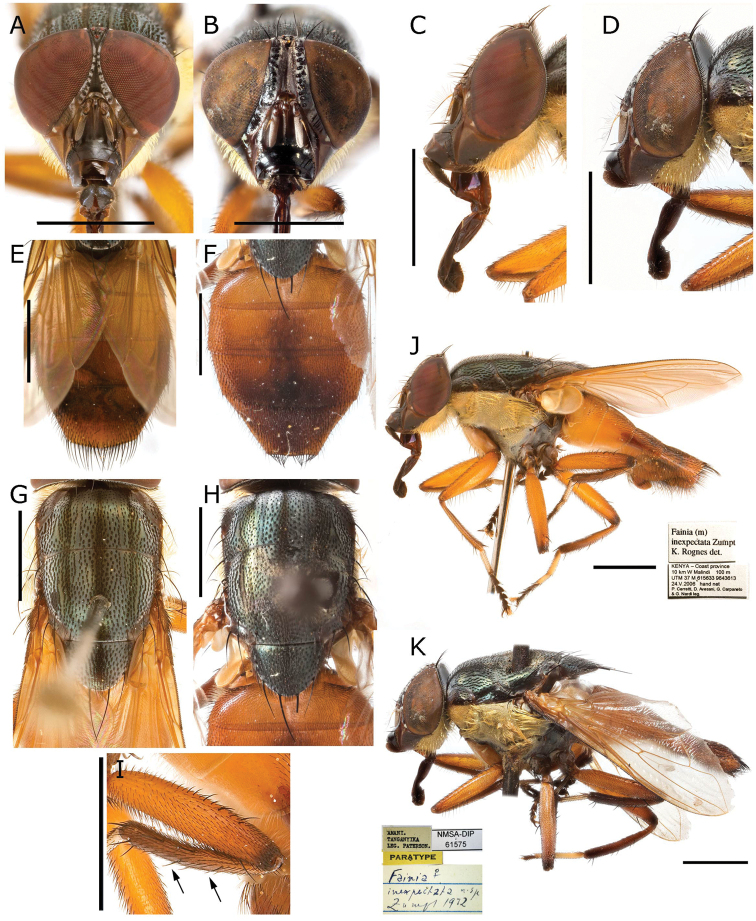
*Fainia inexpectata* Zumpt, 1973. General body views of male (MZSUR) and female (paratype NMSA DIP 61575) **A, C, E, G, I, J** male **A** head in frontal view **C** head in lateral view **E** abdomen in dorsal view **G** thorax in dorsal view **I** hind tibia with two anterodorsal setae (arrows) **J** lateral habitus and labels **B, D, F, H, K** female **B** head in frontal view **D** head in lateral view **F** abdomen in dorsal view **H** thorax in dorsal view **K** lateral habitus and labels. Scale bars: 2 mm.

**Figure 11.**
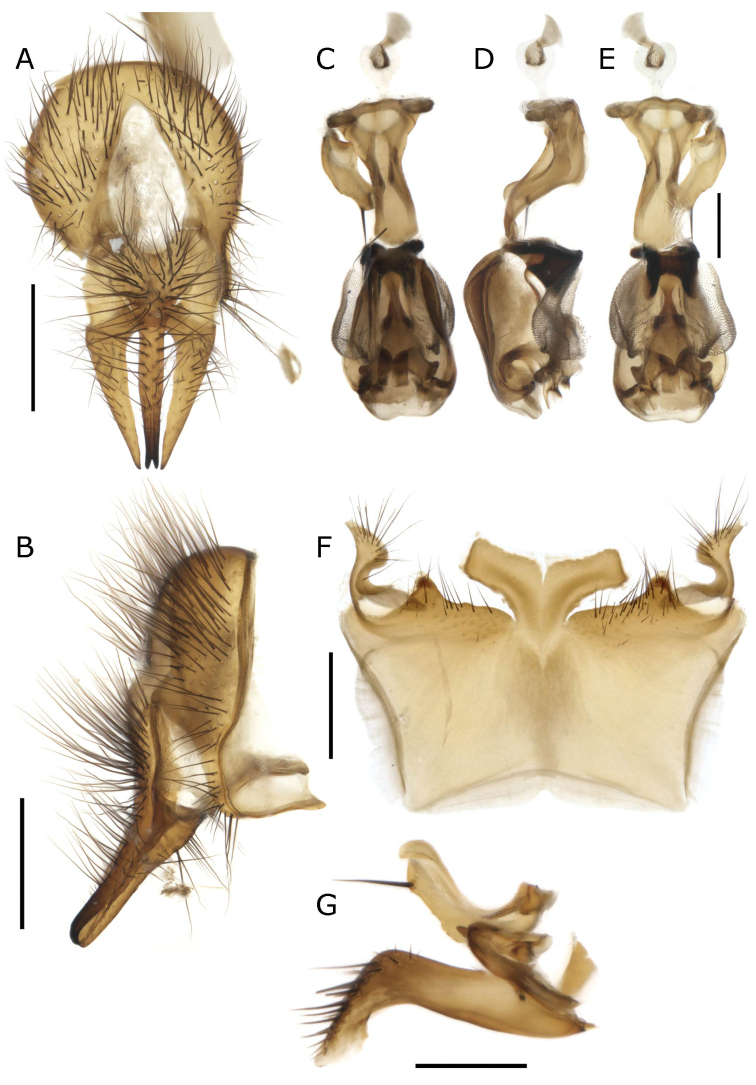
*Fainia inexpectata* Zumpt, 1973. Male terminalia (MZSUR) **A, B** epandrial complex in dorsal (**A**) and lateral (**B**) view **C–E** phallus in dorsal (**C**), lateral (**D**) and ventral (**E**) view **F** sternite 5 in ventral view and **G** postgonite (upper) and pregonite in lateral-internal view (**G**). Scale bars: 0.2 mm.

**Figure 12.**
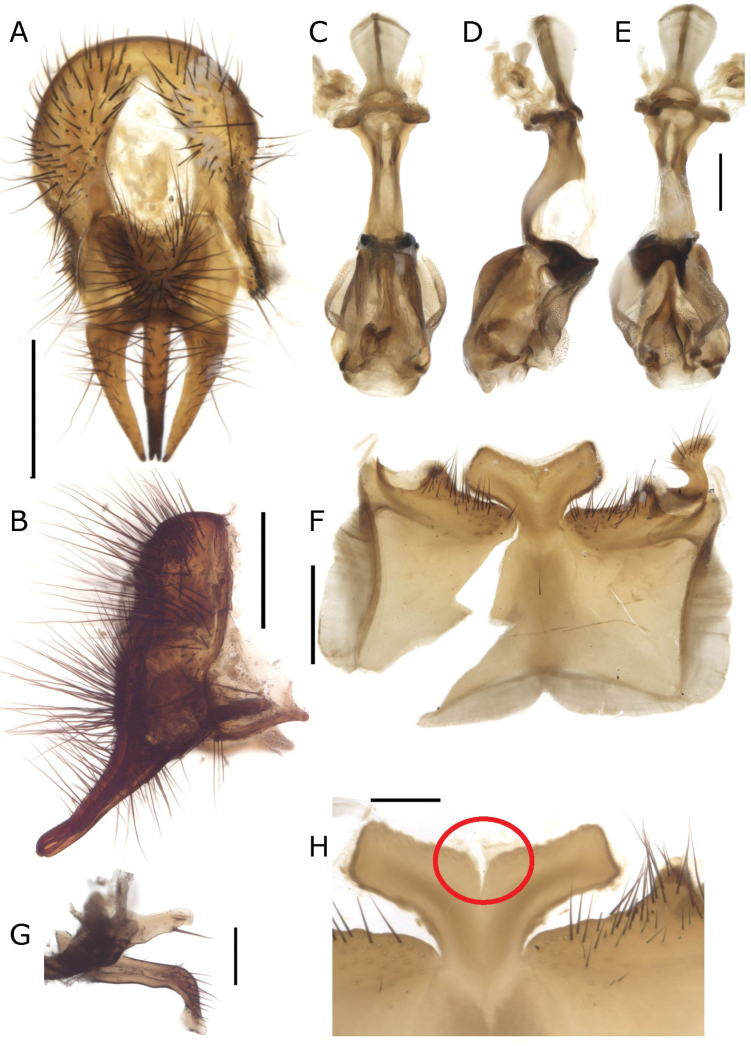
*Fainia kirinyaga* Lehrer, 2007b holotype (SMNHTAU (TAUI) 318989) male terminalia **A, B** epandrial complex in dorsal (**A**) and lateral (**B**) view **C–E** phallus in dorsal (**C**), lateral (**D**) and ventral (**E**) view **F** sternite 5 in ventral view and **G** postgonite (upper) and pregonite lateral-internal view (**G**) **H***Fainia inexpectata* Zumpt, 1973 details of medial lobe tear (red circle) of the sternite 5 in ventral view. Scale bars: 0.2 mm.

**Figure 13.**
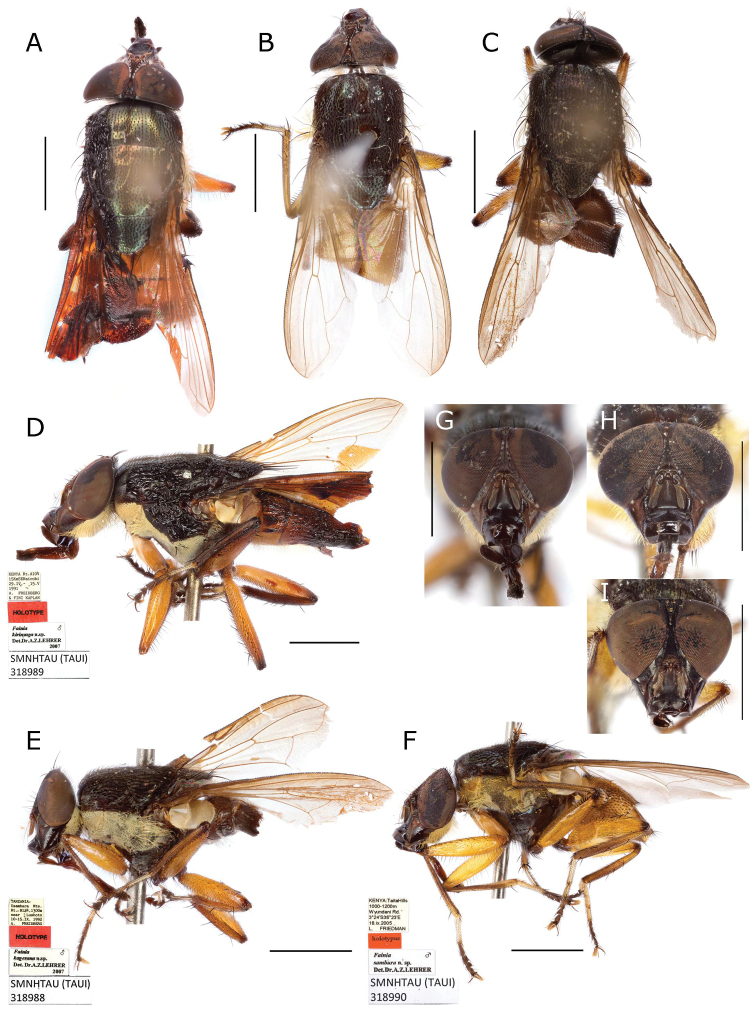
*Fainia kirinyaga* Lehrer, 2007b holotype (SMNHTAU (TAUI) 318989), *Fainia sambura* Lehrer, 2008 holotype (SMNHTAU (TAUI) 318989) and *Fainia kagerana* Lehrer, 2007a nom. nud. (SMNHTAU (TAUI) 318990) general body and heads views **A, D, G***F. kirinyaga* dorsal habitus view (**A**), lateral habitus view (**D**) and head frontal view (**G**) **B, F, H***Fainia sambura* dorsal habitus view (**B**), lateral habitus view (**F**) and head frontal view (**H**) **C, E, I***Fainia kagerana* nom. nud. dorsal habitus view (**C**), lateral habitus view (**E**) and head frontal view (**I**). Scale bars: 2 mm.

♀♂ ***Head*** (Figs 5A–D, 8A–D, 10A–D, 13D–I). Fronto-orbital plate and parafacial ground colour black, covered with silvery microtomentum; parafacial with a glossy black spot; face ground colour black-brown, covered with silvery microtomentum, facial carina protruding (narrow or broad); lower face margin visible in profile, non-rounded, strongly protruding beyond antennal insertion; pedicel and postpedicel ground colour black-brown; arista pectinate, basally yellow and distally dark brown; vibrissa short and thick, 2–4 supravibrissal setulae adjacent to vibrissa; genal dilation anteriorly glossy black and bare, posteriorly covered with dense yellow microtomentum with hairs, generally with tiny piliferous dots around insertion of each hair; occipital area behind postocular setae with a bare and shiny broad black margin. ***Thorax*** (Figs 5G, H, K, L, 8G, K, J, K, 10G, H, J, K, 13A–F). General colouration dark olive green with 3 longitudinal dorsal dark vittae, hair insertions with small piliferous dots; pleura covered by dense yellow microtomentum (in different extension degrees); dorsal chaetotaxy reduced, presutural acrostichal, dorsocentral and intra-alar setae absent and postsutural setae reduced to prescutellars and supra-alar; 2 (outer and anterior) post-postpronotal setae present, postalar wall and suprasquamal ridge bare. ***Wing*** (Fig. 4E). Cell *r_4+5_* always open. ***Legs*** (Figs 5J–L, 8J, K, 10I–K). Femora reddish-yellow; male mid-femur bearing a distal posteroventral row (ctenidium) of closely spaced setae (Fig. 5J) that are spine-like in male but not in female; tibiae yellow to brown; first tarsomeres creamy white; first and second hind tarsomeres creamy, almost white. ***Abdomen*** (Figs 5E, F, K, L, 8E, F, J, K, 10E, F, J, K). Longer than broad, extending to wing tip or even further; colour generally yellow-orange and sometimes partly brown. ***Male terminalia*** (Figs 1–3, 6, 7, 9, 11, 12). Sternite 5 divided into three posterior lobes, 2 outer and 1 median. Outer lobes elongated and posteriorly slender or broad, tending to an inward curve (Figs 3, 6F, 7C, I, 9F, 11F, 12F), with or without abundant setae of different lengths and thickness; medial lobe forming a broad protuberance of varied shape (Figs 3, 6F, 7C, I, 9F, 11F, 12H). Phallus with basi- and distiphallus not fused and connected through a membrane (connection membrane) (Fig. 1A–C); epiphallus absent; basiphallus with two anterolateral processes; ventral plate articulate; paraphallus distally globular.

### Key to *Fainia* species

**Table:** 

1	Thorax with katepisternum partially or completely covered with dense yellow microtomentum, meron with lighter yellow microtomentum (Fig. 10J, K). ♂ Hind tibia with 2 anterodorsal setae and 2 posterodorsal setae **2**	
–	Thorax with katepisternum and meron glossy or covered with a light yellow-silvery microtomentum (Fig. 5K, L). ♂ Hind tibia with 3 anterodorsal setae and 3 posterodorsal setae. ♂ Eyes separated at narrowest point by 1.50 to 2.00 times width of anterior ocellus (Fig. 5A); abdominal sternite 5 with outer lobes covered with thick hairs and median lobe with a rounded, protruding posterior margin (Fig. 6F). ♀ Abdomen with posterior margin of tergite 5 without an emargination and with a row of thin, sparse, black marginal setae (Fig. 5F)	***F. albitarsis* (Macquart, 1846)**
2	Katepisternum completely covered with dense yellow microtomentum, as on anepisternum (Fig. 8J, K). ♂ Eyes separated at narrowest point by 0.75 to 1.30 times width of anterior ocellus (Fig. 8A); abdominal sternite 5 with long outer lobes covered by long hairs, median lobe square with straight posterior margin (Fig. 9F). ♀ Abdomen with posterior margin of tergite 5 with a triangular emargination (inward) and with a row of thick, long, black marginal setae (Fig. 8F)	***F. elongata* (Bezzi, 1908)**
–	Katepisternum not completely covered with dense yellow microtomentum (as on anepisternum), which is restricted to upper half (Fig. 10J, K). ♂ Eyes separated at narrowest point by 1.50 to 2.00 times width of anterior ocellus (Fig. 10A); abdominal sternite 5 with short and curved outer lobes covered by a few thin hairs; median lobe almost triangular (broken or not in the middle of posterior margin) (Figs 11F, 12F, H). ♀: Abdomen with posterior margin of tergite 5 without an emargination and with a row of thick, short, black marginal setae (Fig. 10F)	***F. inexpectata* Zumpt, 1973**

### Fainia albitarsis

Taxon classificationAnimaliaDipteraRhiniidae

(Macquart, 1846)

BCC5D471-F1FF-5DE6-85AE-65785166D51E

Figs 1 , 2 , 4 , 5 , 6 , 7 , 13B, C, E, F, H, I

≡ Idia albitarsis Macquart, 1846: 321 (*teste*Zumpt 1958)
= Idia eupoda Loew, 1852: 660 [redescribed 1862: 24] (*teste*Peris 1952; Zumpt 1958)
= Idia extensa Walker, 1858: 211 (*teste*Peris 1952; Zumpt 1958)
= Fainia kagerana Lehrer, 2007a: 2 nom. nud. (no differential diagnosis)
= Fainia sambura Lehrer, 2008: 16 syn. nov.

#### Type localities and repositories of primary types.

*Idia albitarsis*: South Africa, Cafrerie [= KwaZulu-Natal], (?co)Type(s) female(s) in MNHN (destroyed, not in remnants of the Macquart Collection, Thomas-Cabianca, pers. obs., lateral head view illustrated in Macquart 1846: plate 17, figure 2). *Idia eupoda*: Mozambique, Inhambane, (?co)Type(s) [female(s)] in ZMHB (number of type specimens not specified, not located, considered missing, Thomas-Cabianca, pers. obs.; sex and locality specified in Loew (1862: 24)). *Idia extensa*: South Africa, Port Natal [= Durban], (?co)Type(s) male(s) in NHMUK (Natural History Museum UK) (number of type specimens not specified, not examined). *Fainia sambura*: Kenya, Taita Hills, male HT in SMNHTAU (TAUI) (examined).

#### Distribution.

Central African Republic, Democratic Republic of the Congo, ?Ghana, Kenya, Malawi, Mozambique, Namibia, Sierra Leone, South Africa, Sudan, Tanzania, Uganda, Zimbabwe (Peris 1952, 1956; Zumpt 1958; Pont 1980; Kurahashi and Kirk-Spriggs 2006; Lehrer 2011).

#### Biology.

Ecology, immature stages and life history unknown.

#### Redescription

**(male and female).** Length 7.83 mm [6.55–9.00 mm] (n = 9). ***Head*** (Fig. 5A–D). ***Thorax*** (Fig. 5G, H, K, L). Chaetotaxy: acrostichal setae = 0 + 1, dorsocentral setae = 0 + 1, intra-alar setae = 0 + 1, postpronotal lobe setae = 1 long and sometimes 1 extra short, outer post-postpronotal lobe setae present, presutural seta present, supra-alar setae = 2, marginal scutellar setae = 3, discal scutellar setae = 0, proepisternal setae = 2, proepimeral seta = 0. Katepisternum covered with light silvery microtomentum; proepimeron, proepisternum, anepimeron, anepisternum and inferior half of postpronotal lobe covered with dense yellow microtomentum (Fig. 5K, L), anepisternal setae = 2 anterior to an extra posterior dense row of yellow hairs (Fig. 5K, L). ***Wing*** (Fig. 5K, L). Tegula and basicosta black-brown, outer margin along costal vein lightly infuscated. Lower calypter yellow and slightly longer than broad. ***Legs*** (Fig. 5K, L). Femora yellow-orange, tibiae yellow to brown. ***Abdomen*** (Fig. 5E, F, K, L). Yellow-orange, longer than broad. **Male** (n = 8). ***Head*** (Fig. 5A, C). Eye bare, inner facets moderately enlarged, but not demarcated from outer ones. Eyes separated by 0.06 times width of head [0.05–0.06] (at narrowest point, one-half to two times width of anterior ocellus); eye length 2.99 times height of gena [2.70–3.16]. Postpedicel length 2.39 times length of pedicel [2.10–2.27]; ocellar setae well-developed, inner vertical seta present, outer vertical seta absent; 6–8 frontal setae; palpus width around 2 times width of postpedicel in broadest area. ***Legs*.** Fore tibia 1–2 anterodorsal setae; mid-tibia 1 anterodorsal seta, 1 posterodorsal seta; hind tibia 2 anterodorsal setae, 2 posterodorsal setae, 2 anteroventral setae. ***Abdomen*.***Terminalia* (Fig. 6). Median lobe width 0.33 times the width of sternite 5, posterior margin round with a lighter and less sclerotised margin; section that connects with outer lobes covered with scattered black hairs. Outer lobes shorter and broader than in *F. elongata* (Fig. 9F), terminal area globular and covered with long and thick black setae, surrounded by a lighter halo with dense yellow vestiture. Surstylus wide and rectangular (plate form), slightly curved outward in medial distal edge (Fig. 6A), posterior edge serrated and grooved (Figs 6A, 7G); ventrally and dorsally covered with black hairs in medial area. Cercus slender and fused, with long black setae, apically bifurcated (Figs 6A, 7G) forming an inward hook in lateral view (Fig. 7H). Phallus as Figs 6C–E, 7D, E, ventral plate in ventral view M-shaped (which is obvious (Figs 1C, 6E) or not, depending of ventral plate position); postgonite and pregonite as in Fig. 6G, H. **Female** (n = 1). ***Head*** (Fig. 5B, D). Eyes separated 0.23 times of the head width at the narrowest point; eye length 4.14 times gena height; postpedicel 2.21 times pedicel length; proximal edge of fronto-orbital plate weakly concave towards frontal vitta; fronto-orbital plate 0.58 times frontal vitta width at ocellar triangle tip; ocellar setae well-developed and proclinate, 7–8 frontal setae, 2 proclinate orbital setae, 1 reclinate orbital seta; palpus width more than 3.00 times postpedicel width in broadest area. ***Legs*.** Fore tibia 2 anterodorsal setae; mid-tibia 1 anterodorsal seta, 1 posterodorsal seta, 1 anteroventral seta, 2 posteroventral setae; hind tibia 2 anterodorsal setae, 1–2 posterodorsal setae, 2 anteroventral setae. ***Abdomen*** (Fig. 5F, L). Posterior margin of tergite 5 without emargination, marginal setae thin and black.

#### Discussion.

*Fainia albitarsis* is widely distributed in the Afrotropical Region. It was adequately diagnosed by Peris (1952) and redescribed by Zumpt (1958), but the illustrations of sternite 5 are incongruent between the two authors. Photographs of sternite 5 (Fig. 6F) are provided here for a proper determination of the species. The HT or STs were found to be destroyed by pests at MNHN. Additionally, the HT or STs of *F. eupoda* were not found in ZMHB and we consider it missing. The specimen assigned as HT of *Idia eupoda* in ZMHB is labelled as ‘*Pr. b. sp* Krebs // 4532 // Type (red-label) // *eupoda* Loew*’; ‘*Pr. b. sp*’ refers to *Promontorium bonae spei*, Latin for “Cape of Good Hope’’ in South Africa and it was collected by Ludwig Krebs (1792-1844), Cape naturalist to the King of Prussia. This differs from the published type locality and collector: Inhambane, Mozambique leg. Peter, suggesting that the specimen is, in fact, not a type. Specimens from Democratic Republic of the Congo, Kenya and South Africa (see material examined section) identified by Knut Rognes, together with the description of Zumpt (1958), were used for the proper determination of this species. As the descriptions were adequate for identification, neotypes are not required for *Fainia albitarsis* or *Idia eupoda*. The synonymy of *Idia extensa* was first published by Peris (1952), and seems reliable.

The description of *Fainia kagerana* nom. nud. is an invalid nomenclatural act in terms of ICZN Article 13.1.1 because it lacks a comparative diagnosis. In addition, the descriptions and drawings of *F. kagerana* nom. nud. (Lehrer 2011: 59–61) (Fig. 13C, E, I) and *F. sambura* syn. nov. (Lehrer 2011: 63–65) (Fig. 13B, F, H) match the morphology of *F. albitarsis.* On examining the *F. sambura* syn. nov. HT, including the male terminalia (dissected by Lehrer) (Fig. 7), we found that the surstyli, cerci and ventral plate exhibit the same diagnostic characters as described above (Fig. 6A, B). Careful examination of the ventral plate of the phallus revealed that the structure is articulated, with the joint located within the basi- and distiphallus membranous connection. This articulation can produce different orientations of the ventral plate in lateral and ventral views of the phallus, obscuring their typical ‘M’ shape visible in ventral view in various specimens. The phallus drawings of *F. kagerana* nom. nud. (Lehrer 2011: fig. 36D) and *F. sambura* syn. nov. (Lehrer 2011: fig. 39D) show different orientations of the ventral plate in lateral view, suggesting that they could be different species. The ‘M’ shape of the ventral plate can be clearly observed in the specimen of *F. kagerana* nom. nud. (Figs 6E, 7E) and partially observed in the HT of *F. sambura* syn. nov. as it was partially damaged (it was crushed between the lid and wall of the microvial) (Fig. 7J). Based on this evidence, we conclude that *F. sambura* is synonym of *F. albitarsis* and *F. kagerana* nom. nud. corresponds to *F. albitarsis*.

#### Type material examined.

*Fainia sambura*HT. 1 ♂ KENYA: Taita Hills / 1000–1200 m / Wyundani Rd. 3°24'S, 38°23'E / 18.ix.2005 / L. FRIEDMAN // holotypus // n. sp. / det. Dr A.Z. Lehrer // SMNHTAU (TAUI) 318990.

#### Other material examined.

19 specimens (10 ♀♀ 9 ♂♂).

**Democratic Republic of the Congo – Katanga** • 1 ♂; Ubani Valley Umbombo Dist.; Mar. 1915; Yoppin leg., det. Thomas-Cabianca, A., 2018; DMSA DIP 6260. – **Oriental** • 1 ♀; Bomane village area; 01°16.283'N, 23°43.994'E; 24 May 2010; Kirk-Spriggs, A.H. leg.; lowland evergreen second dry forest; Malaise trap; det. Rognes, K., 2012; BMSA-BECE 01314.

**Kenya – Coast** • 1 ♀; N. edge of Arabuko Sokoke Forest; UTM 37 M 607257 9644873, 83 m elev.; 28 May 2006; Avesani, D., Carpaneto, G., Nardi, G. & Cerretti, P. leg.; hand net; with larva, det. Rognes, K.; MZSUR – **Nairobi** • 1 ♀; Kakura Forest; 01°14'28.64"S, 36°49'54.97"E; 1672 m elev.; 21–23 Nov. 2017; PINDIP-Course leg.; Kenyan dry forest; 6 m elev. Malaise trap; det. Thomas-Cabianca, A., 2017; CEUA, DNA-COI USA04 • 1 ♂, same collection data as previous; 23 Nov. 2017; general sweeping; CEUA, DNA-COI USA03.

**South Africa** – **KwaZulu-Natal** • 1 ♂; Amatigulu Nature Reserve, north of Tugela River mouth; 29°12'S, 31°36'E; 25–26 Aug. 2006; Davies, G.B.P. leg.; caught hovering in group; det. Thomas-Cabianca, A., 2018; NMSA DIP 84325 • 1 ♀; Durban; 1914; Haygarth, W.J. leg.; det. Villeneuve (as *Idiella eupoda*); SAMC DIP A015193 • 1 ♂; Manguzi Forest Reserve; 26°59'32"S, 32°43'25"E; 61 m elev.; 13–17 Dec. 2010; Kirk-Spriggs, A.H. leg.; indigenous sand forest; Malaise trap; det. Rognes, K., 2012; BMSA (D) 30066 • 1 ♀; Ndumo Game Reserve, Red Cliffs/Shokwe area at Ingwavuma; 26°52.125'S, 32°13.731'E; 30 Nov.–04 Dec. 2009; Kirk-Spriggs, A.H. leg., *Ficus* forest; Malaise trap; det. Rognes, K., 2012; BMSA (D) 15187 – **Mpumalanga** • 1 ♀ 1 ♂; Blyde River, Burkes Luck; 24°40'30"S, 30°48'40"E; 1200 m elev.; 24 Dec. 1990; Roth, V. & Roth, B. leg.; det. Thomas-Cabianca, A., 2018; NMSA DIP ♀: 84327 ♂: 84332 – **Western Cape** • 2 ♂♂; George (Caplant); 01 Feb. 1918; Brauns, Dr H. leg.; det. Thomas-Cabianca, A., 2018 (previously determined as *Rhinia apicalis* in the collection); NMSA DIP 020015 • 1 ♀; *Pr. B. sp.*; Krebs leg.; det. Loew (previously determined as *Fainia eupoda* in the collection); ZMHBHT 4532.

**Tanzania – Morogoro** • 1 ♀; Udzungwa Mountains National Park, Mito Mitato Plot 13; 674 m elev.; 26 Oct. 2014; Malumbre-Olearte, J. leg.; det. Thomas-Cabianca, A., 2019; ZMUC, DNA-COI K3 • 1 ♀ 2 ♂♂; Udzungwa Mountains National Park, Mizimu Camp.; 769 m elev.; 01 Sep. 2013; Pape, T. & Scharff, N. leg.; det. Thomas-Cabianca, A., 2019; ZMUC, ♀: DNA-COI K7, ♂♂ DNA-COI K6 K20 – **Tanga** • 1 ♂; Usambara, Mts., Rt. B124, 1300 m elev., near Lushoto; 10–15 Sep. 1992; Freidberg, A. leg; det. Thomas-Cabianca, A. 2019; (previously determined as *Fainia kagerana*HT by Dr A.Z. Lehrer, 2007 in the collection); SMNHTAU (TAUI) / 318988.

### Fainia elongata

Taxon classificationAnimaliaDipteraRhiniidae

(Bezzi, 1908)

AA24325C-43D2-5831-A952-BF5EBC4D661C

Figs 3 , 8 , 9

≡ Stomatorrhina elongata Bezzi, 1908: 383 (*teste*Zumpt 1958)
= Idiella major Malloch, 1926: 510 (*teste*Peris 1952; Zumpt 1958)

#### Type localities and repositories of primary types.

*Stomatorrhina elongata*: Bas-Congo (= Democratic Republic of the Congo), male HT in IRSNB (Royal Belgian Institute of Natural Sciences, Brussels, Belgium) (description based on a single male specimen, not examined). *Idiella major*: Sierra Leone, Masimera to Yonnibanna, (?co)Type(s) female(s) in NHMUK (Natural History Museum UK) (number of type specimens not specified, locality specified in Peris (1952: 48), not examined).

#### Distribution.

Cameroon, Central African Republic*, Democratic Republic of the Congo, Equatorial Guinea, Ivory Coast, Kenya, ?Madagascar, Malawi, Mozambique, Namibia, ?Nigeria, Rwanda, Sierra Leone, South Africa, Sudan, Tanzania, Togo, Uganda, Zimbabwe (Malloch 1926; Peris 1952, 1956; Zumpt 1958, 1962; Pont 1980; Kurahashi and Kirk-Spriggs 2006).

#### Biology.

Ecology, immature stages and life history unknown.

#### Redescription.

Length 10.76 mm [10.60–10.87] (n = 3) ***Head*** (Fig. 8A–D). ***Thorax*** (Fig. 8G–K). Acrostichal setae = 0 + 1, dorsocentral setae = 0 + 1, intra-alar setae = 0 + 1, post postpronotal lobe setae = 1 long and 1 short, outer post postpronotal lobe seta present, supra-alar setae = 2, marginal scutellar setae = 3, discal scutellar setae = 0, proepisternal setae = 2, proepimeral seta = 0. Proepimeron, proepisternum, anepimeron, anepisternum, katepisternum and inferior half of postpronotal lobe covered with dense yellow microtomentum (Fig. 8J, K); meron also covered, but with microtomentum lighter, anepisternal setae = 2 anterior to an extra dense row of yellow hairs (Fig. 8J, K). ***Wing***. Tegula and basicosta black-brown, outer margin along costal vein light infuscate, lower calypter yellow and slightly longer than broad. ***Legs*** (Fig. 8J, K). Femora yellow, tibiae yellow to brown. ***Abdomen*** (Fig. 8E. F). Yellow-orange, longer than broad. **Male** (n = 2). ***Head*** (Fig. 8A, C). Eye bare, inner facets moderately enlarged but not demarcated from outer ones. Eyes separated by 0.04 times width of head [0.04–0.04] (at narrowest point between 1.10 to 1.30 times anterior ocellus width); eye length 3.51 times height of gena [3.49–3.54]. Postpedicel length 2.52 times length of pedicel [2.44–2.61], ocellar setae well-developed, inner vertical seta present, outer vertical seta absent, 8-10 frontal setae, palpus width in broadest area around 2.50 times width of postpedicel. ***Thorax*** (Fig. 8G, J). ***Legs*** (Fig. 8J). Fore tibia with 2–3 anterodorsal setae; mid-tibia with 1 anterodorsal seta, 1 posterodorsal seta; hind tibia with 3 anterodorsal setae (row-like), 3 posterodorsal seta (row-like), 2 anteroventral setae. ***Abdomen*** (Fig. 8E, J). ***Terminalia*** (Fig. 9). Medial lobe 0.5 times width of sternite 5, posterior margin straight or almost straight and less sclerotised, area that connects with outer lobes densely covered by black hairs (Fig. 9F). Outer lobes longer and narrow, curved in proximal direction (like an open ‘C’), terminal area round, covered by long and thick black setae, surrounded by a lighter halo with yellow vestiture (Fig. 9F). Sustylus rectangular (Fig. 9A, B), thinner and more slender than in *F. albitarsis* (Fig. 6A, B), posterior area darker (Fig. 9A, B), ventrally and dorsally with black setae (Fig. 9A, B), in lateral view (Fig. 9B) slightly curved inwards at ventral posterior region and proximally pointed (Fig. 9B). Cercus slender and fused, covered with long black setae, forming an inward hook apically (Fig. 9B). Phallus as in Fig. 9C–E, ventral plate in ventral view as in Fig. 9E; postgonite and pregonite as in Fig. 9C–E, G. **Female** (n = 1). ***Head*** (Fig. 8B, D). Eyes separation 0.20 times width of head, eye length 3.12 times height of gena. Postpedicel 2.08 times length of pedicel; frontal vitta subparallel-sided; fronto-orbital plate 0.60 times as wide as frontal vitta at tip of ocellar triangle; ocellar setae well-developed and proclinate, 11 frontal setae, 5 or more proclinate orbital setae, 1 reclinate orbital seta; palpus width more than 2.00 times postpedicel width in broadest area. ***Thorax*** (Fig. 8H, K). ***Legs*** (Fig. 8K). Fore tibia 2 anterodorsal setae; mid-tibia 1 anterodorsal seta, 1 posterodorsal seta, 1 anteroventral seta, 2 posteroventral setae; hind tibia 2 anterodorsal setae, 2 posterodorsal setae, 2 anteroventral setae. ***Abdomen*** (Fig. 8F, K). Tergite 5 with a triangular middle incision (Fig. 8F).

#### Discussion.

We were not able to examine the type material of *Fainia elongata* or *Idiella major*, but *F. elongata* is a well-defined species, properly described by Bezzi (1908) and diagnosed by Peris (1952) and Zumpt (1958). The synonymy was first published by Peris (1952) and seems reliable.

#### Material examined.

21 specimens (7 ♀♀ 14 ♂♂).

**Cameroon** • 1 ♂; Páma-Quelle, Lobaje, Marsch am-Ubangi (Neu-Kamerun); 1913; Ramsay, S.G. leg.; det. Zumpt, F., 1955; ZMHB Dipt S06219 TS no. 19 • 1 ♂; Páma-Quelle (Neu-Kamerun); 1913; Ramsay, S.G. leg.; det. Zumpt, F., 1955; ZMHB Dipt S06219 TS no. 3; • 1 ♀ 2 ♂♂; Páma-Quelle, Lobaje // Marsch am-Ubangi (Neu-Kamerun); 15 Feb. 1913; Ramsay, S.G. leg.; det. Thomas-Cabianca, A., 2019; ZMHB Dipt ♀: S06217 ♂♂: S06219 • 1 ♂; Páma-Quelle // Mboko (Neu-Kamerun); 23 Feb. 1913; Ramsay, S.G. leg.; ZMHB Dipt S06219.

**Central African Republic – Sangha-Mbaéré** • 5 ♂♂; Parc National de Dzanga-Ndoki, Mabéa Bai, 21.4 Km 53'NE Bayanga; 3°02.01'N, 16°24.57'E; 510 m elev., 03–04 May 2001; van Noort, S. leg.; marsh clearing; lowland rainforest; Malaise trap; det. Thomas-Cabianca, A., 2018; SAMC DIP A015267 • 1 ♂; Parc National de Dzanga-Ndoki, 38.6 km 173'S Lidjombo; 2°21.60'N, 16°09.20'E; 350 m elev.; 21–22 May 2001; van Noort, S. leg.; lowland rainforest; Malaise trap; det. Thomas-Cabianca, A., 2018; SAMC • 1 ♀ 2 ♂♂; same collection data as previous; 23–24 May 2001; SAMC DIP ♀: A015266; ♂: A015269.

**Democratic Republic of the Congo – Oriental** • 1 ♀; Yafira Forest transect; 0.70269'N, 24.20080'E; 29–31 May 2010; Kirk-Spriggs, A.H. leg., primary lowland evergreen forest; Malaise trap; det. Rognes, K., 2012; BMSA-BECE 03118 • 1 ♀ 1 ♂, same collection data as previous; 0.70269'N, 24.20107'E, lowland evergreen swamp forest; BMSA-BECE ♀: 03372 DNA-COI F2, ♂: 03371 DNA-COI F5.

**Equatorial Guinea** • 1 ♀; Uelleburg. Benito Mts. (Spanish Guinea); 1–14 Feb. 1908; Tessmann, S.G. leg.; ZMHB Dipt S06219.

**South Africa – KwaZulu-Natal** • 1 ♀; Ramsgate Butterfly Sanctuary; 30°53.3'S, 30°20.4'E; 26–29 Apr. 2004; Mostovski, M. leg.; light trap; det. Thomas-Cabianca, A., 2018; NMSA DIP 84387.

**Zimbabwe** • 1 ♀; Bomponi, Vumba; 28 Jul. 1965; Cookson, D.M. leg.; det. Zumpt, F., 1969; NMSA DIP 019870.

### Fainia inexpectata

Taxon classificationAnimaliaDipteraRhiniidae

Zumpt, 1973

4B39DA06-DE9C-545E-846B-A07E5ADBEDD6

Figs 10 , 11 , 12 , 13A, D, G

Fainia inexpectata Zumpt, 1973: 157
= Fainia kirinyaga Lehrer, 2007b: 2 syn. nov.

#### Type localities and repositories of primary types.

*Fainia inexpectata*: Ivory Coast, Lamto, male(s) HT and PTs in MNHN (examined); Tanzania, Amani, male and female PTs in NMSA (examined). *Fainia kirinyaga*: Kenya, Nairobi, male HT in SMNHTAU (TAUI) 318989 (examined).

#### Distribution.

Ivory Coast, Kenya, Malawi*, Tanzania (Zumpt 1973; Pont 1980; Lehrer 2007b).

#### Biology.

Ecology, immature stages and life history unknown.

#### Redescription.

A proper and complete description with male terminalia illustrations was given by Zumpt (1973). Here, we provide additional diagnostic characters, based on measurements and discuss the sternite 5 shape. Length 10.56 mm [10.14–11.13 mm] (n = 4). **Male** (n = 2). ***Head*** (Figs 10A and C). Eyes separated by 0.05 times width of head [0.04–0.05] (at narrowest point around 1.75 times the width of anterior ocellus); eye length 2.99 times height of gena [2.70–3.40]. Postpedicel length 2.28 times length of pedicel [2.09–2.52]. ***Terminalia*** (Figs 11, 12). Sternite 5 posteriorly formed by 3 lobes, 1 median and 2 outers (Figs 11F, 12F). Median lobe as Figs 11F, 12F and H, posterior margin triangular with a middle incision inwards, that could be slightly torn (Figs 11F, 12H) or not (Fig. 11F). Lateral lobes shorter than *F. elongata*, as in Fig. 9F. Surstylus and cercus as Fig. 11A, B. Phallus as in Fig. 11C–E, ventral plate in ventral view as in Fig. 11E; post- and pregonite as in Fig. 11G. **Female.** (n = 1). ***Head*** (Fig. 10B, D). Eyes separated by 0.20 times width of head; eye length 3.93 times height of gena. Postpedicel length 2.46 times pedicel length; fronto-orbital plate 0.70 as wide as frontal vitta at tip of ocellar triangle.

#### Discussion.

*Fainia inexpectata* is an uncommon Afrotropical species. The male terminalia were dissected by Zumpt and are preserved in a slide mounting preparation. The preserved terminalia are squashed and the structures overlap, so it was impossible to make a proper examination. Thus, the male terminalia structures were recognised and identified using a drawing provided by Zumpt (1973: fig. 4).

The description and drawings of *F. kirinyaga* syn. nov. (Lehrer 2011: 62–63) (Figs 12, 13A, D, G) match with *F. inexpectata.* After reviewing the HT of *F. kirinyaga* syn. nov., including the male terminalia (dissected by Lehrer and preserved in a microvial) (Fig. 12A–G), we conclude that the specimen belongs to *F. inexpectata*. We observed an apparent difference in the posterior area of the median lobe of sternite 5, which in *F. kirinyaga* syn. nov. (Fig. 12F) is continuous and, in *F. inexpectata* (Figs 11F, 12H and Zumpt 1973: fig. 4), apparently has a mid-ventral incision. After a careful examination under the microscope, we concluded that this incision is a tear in the structure since it is not surrounded by membrane (Fig. 12H).

#### Type material examined.

*F. inexpectata*HT and PT: 4 ♂ Ivory Coast, Lamto / v. 1971, leg. D. Lachaise // det. Zumpt, 1973. At MNHN • *F. inexpectata*PT: 1 ♀ // PARATYPE // Amani, Tanganyika [= Tanzania] / leg. Paterson // det. Zumpt 1973 // NMSA DIP 61575 • *F. inexpectata*PT: 1 ♂ // PARATYPE // Amani, Tanganyika [= Tanzania] / leg. Paterson // Slide no 20 // det. Zumpt 1973 // NMSA DIP 61575 • *Fainia kirinyaga*HT: 1 ♂ KENYA Rt. A104 / 15 km SE Nairobi / 29.iv.-15.v / 1991 / A. FREIDBERG / & FINI KAPLAN // HOLOTYPE // n. sp / det. Dr A.Z. Lehrer / 2007 // SMNHTAU (TAUI) 318989.

#### Other material examined.

9 specimens (6 ♀♀ 3 ♂♂).

**Kenya – Coast** • 1 ♂; 10 km W. Malindi; UTM 37 M 615633 9643613; 100 m elev.; 24 May 2006; Cerretti, P., Avesani, D., Carpaneto, G. & Nardi, G. leg.; hand net; det. Rognes, K.; MZSUR, DNA-COI F6.

**Malawi – Mulanje** • 1 ♀; Mulanje mnt.; 15°56'10"S, 35°31'12"E; 1061 m elev.; 12–14 Nov. 2016; Kirk-Spriggs, A.H. & Muller, B. leg.; stream bed miombo woodland; Malaise traps; det. Thomas-Cabianca, A., 2019; BMSA (D) 92318.

**Tanzania – Iringa** • 1 ♀; Mufindi Dist. Uzungwa Scarp Forest Res.; 750 m elev.; 8–10 Mar. 1996; Mckamey, S. et al. leg.; ZMUC, Canopy light-trapping project; det. Rognes, K., 2013; ZMUCKR 001896, DNA-COI F19 – **Ludewa** • 1 ♀; Nyassa-See, Langenburg; Apr. 1899; Fülleborn, S. leg.; det. Thomas-Cabianca, A., 2019; ZMHB Dipt S06219 (previously determined as *F. albitarsis* by Enderlein, 1919; previously determined as *F. elongata* by Zumpt, 1953) • 1 ♀; Nyassa-See, Langenburg; 22 Nov.– 07 Dec. 1898; Fülleborn, S. leg.; det. Thomas-Cabianca, A., 2019; ZMHB Dipt S06219 (previously determined as *F. elongata* by Zumpt, 1953). – **Tanga** • 1 ♂; East Usambara, Amani, at Sigi River; 500 m elev.; 7 Feb. 1977; Enghoff, H., Lomholdt, O. & Martin O. leg.; det. Rognes, K., 2013; ZMUC 00516250 KR 001894, 00516251 KR 001895 • 2 ♀♀ 1 ♂; Tanga, Mkulumuzi, Gorge, Section No: VII, Tray No.: 8, Jar No. 19; 5–50 m elev.; Mar. 1992; Frontier-ZMUC leg.; det. Thomas-Cabianca, A., 2019; ZMUC.

### Rhinia giriama

Taxon classificationAnimaliaDipteraRhiniidae

(Lehrer, 2007b) comb. nov.

03A9512D-ABD4-5FA6-9C6D-42187474679E

Fig. 14

≡ Fainia giriama Lehrer, 2007b: 3

#### Type locality and repository of primary types.

*Fainia giriama*: Kenya, HT in SMNHTAU (TAUI) 318987 (examined).

#### Distribution.

Kenya (Lehrer 2007b).

#### Biology.

Ecology, immature stages and life history unknown.

#### Discussion.

**Figure 14.**
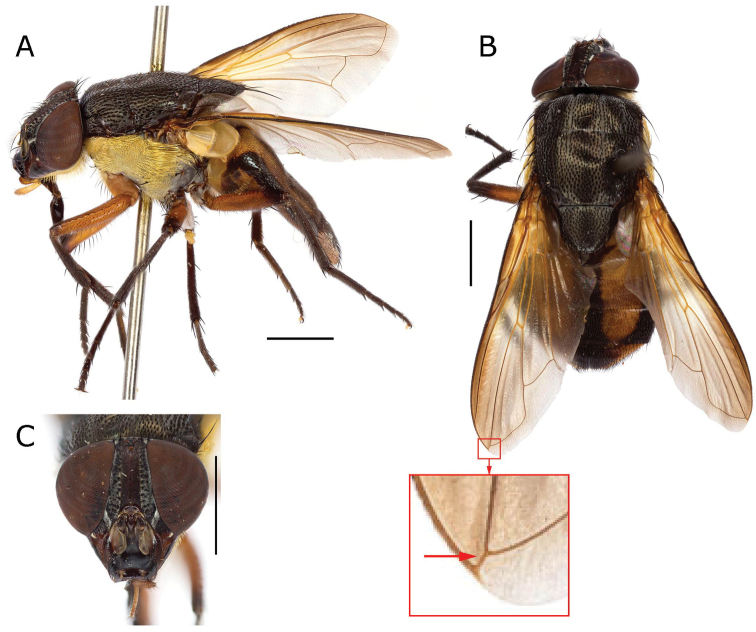
*Rhinia giriama* Lehrer, 2007b comb. nov. holotype (SMNHTAU (TAUI) 318987) general body and head views **A** lateral habitus view **B** dorsal habitus view and details of cell *r*_4+5_ showing long petiole (red square and arrow) and **C** head frontal view. Scale bars: 2 mm.

This is the only species described by Lehrer in *Fainia* that was based on a single female specimen. After examining the HT of *F. giriama* (Fig. 14), we conclude that it belongs to the genus *Rhinia*. The specimen is characterised by having wing cell *r*_4+5_ closed with a long petiole and apical area darkened, fore and mid first tarsomeres dark and palpi long, narrow and uniform in width, generally yellow (Fig. 14A, C). These characters fit the concept of the genus *Rhinia* (Zumpt 1958; Peris 1992) and not *Fainia* (see diagnosis above).

#### Type material examined.

*Fainia giriama*HT: 1 ♀ KENYA Tambach / 40 km E Eldoret / 12.v.1991 / A. FREIDBERG / & FINI KAPLAN // HOLOTYPE // *Fainia* / *giriama* n. sp / det. Dr A. Z. Lehrer / 2007 // SMNHTAU (TAUI) 318987.

##### Notes on Rhiniidae classification and potential apomorphies for Rhiniinae

Brauer and von Bergenstamm (1889) split rhiniids into Cosminidae, Rhininiidae and Rhyncomyiidae. Riley and Johansen (1915) then reclassified them as subfamilies (Cosmininae, Rhininiinae and Rhyncomyiinae) within Calliphoridae. Malloch (1926) classified all rhiniids in Rhiniinae (within Calliphoridae), split into two tribes, based on the proepisternal seta, present in Cosminini and absent in Rhiniini. Malloch’s classification was also followed by Senior-White et al. (1940), but Peris (1952) discarded it, arguing that some species of *Stegemosa* Loew (Cosmininae) lack a proepisternal seta, while some species of *Chlororhina* Townsend (Rhiniinae) present it. Other authors classified Rhiniinae (within Calliphoridae) without using tribes or subfamilies, because of the lack of diagnostic characters and morphological studies (Dear 1977; James 1977; Rognes 1998) or because they considered the subdivisions unnecessary for a higher taxon with so few genera (Peris 1952, 1992). Lehrer (1970) proposed a radical approach, dividing Rhiniinae (within Calliphoridae) into six tribes, based on the morphology of the male terminalia (Isomyiini, Rhiniini, Rhyncomyiini, Stegosomini, Sokotrini and Trychoberiini) and, years later, split rhiniids into three subfamilies: Fainiinae, Rhiniinae and Stomorhiniinae (sic) (Lehrer 2011).

More recently, in addition to the traditional characters used to split the two primary lineages of Rhiniidae (Peris 1952; Zumpt 1958; Kurahashi and Kirk-Spriggs 2006), Fang and Fan (1988) incorporated characters of the phallus. In Cosmininae, the acrophallus is often connected with the base of the hypophallus (= mid-ventral wall) and the epiphallus is developed, while in Rhiniinae, the acrophallus stretches out from the paraphallus and the epiphallus is undeveloped.

Recent molecular evidence, based on DNA Ultra Conserved Element (UCE) sequence data, reconstructed three major clades within Rhiniidae, with Cosmininae split into two clades (one containing the exclusive Oriental genus *Sumatria* and the other, the rest of the Cosmininae genera) and Rhiniinae monophyletic (Buenaventura et al. 2020). In our examination of all of the Afrotropical rhiniids, two morphological characters in the phallus support potential synapomorphies for the Rhiniinae (Table 1). First, the absence of an epiphallus is apomorphic in Rhiniinae, as was also suggested by Fang and Fan (1988) and the epiphallus is present (pleisomorphic state) in other Rhiniidae and its sister group Bengaliinae (Calliphoridae) (Rognes 2009; Cerretti et al. 2019; Kutty et al. 2019; Buenaventura et al. 2020). Second, the basi- and distiphallus are connected by a desclerotised membrane, which is apomorphic in Rhiniinae, whereas they are plesiomorphically fused in other Rhiniidae and Bengaliinae (Rognes 2009).

**Table 1.** Proposed apomorphies (in bold) for Rhiniinae, polarised using the character state found in Bengaliinae (Diptera: Calliphoridae) (Rognes 2009) and Afrotropical Cosmininae (Buenaventura et al. 2020).

Character	Character state		
	Bengaliinae	Cosmininae	Rhiniinae
**Epiphalus**	present	present	**absent**
**Basi- and distiphallus**	fused	fused	**not fused, connected by desclerotised membrane, giving independent mobility to these structures**

## Supplementary Material

XML Treatment for Fainia

XML Treatment for Fainia albitarsis

XML Treatment for Fainia elongata

XML Treatment for Fainia inexpectata

XML Treatment for Rhinia giriama
